# Integrated virulence–resistance profiling of *Helicobacter pylori* reveals context-dependent pathogenic signatures in gastric cancer

**DOI:** 10.3389/fcimb.2026.1812371

**Published:** 2026-05-19

**Authors:** Oliver Mbuthia, Azhar Mehmood, Ishrat Mahjabeen, Mahmood A. Kayani, Jael Obiero, Takashi Matsumoto, Kefa Bosire, Marianne W. Mureithi

**Affiliations:** 1 Department of Medical Microbiology and Immunology, College of Health Sciences, University of Nairobi, Nairobi, Kenya; 2 Department of Biosciences, COMSATS University Islamabad, Islamabad, Pakistan; 3 Department of Reproductive Health and Biology, Kenya Institute of Primate Research, Karen, Nairobi, Kenya; 4 Department of Environmental and Preventive Medicine, Faculty of Medicine, Oita University, Yufu, Japan; 5 Department of Pharmacy and Pharmacognosy, College of Health Sciences, University of Nairobi, Nairobi, Kenya; 6 KAVI – Institute of Clinical Research, College of Health Sciences, University of Nairobi, Nairobi, Kenya

**Keywords:** antibiotic resistance (AMR), gastric cancer, *Helicobacter pylori*, k-means clustering, molecular profiling, transcriptional plasticity, tumor microenvironment, unsupervised clustering

## Abstract

**Background:**

*Helicobacter pylori* contributes to gastric carcinogenesis through diverse virulence mechanisms, transcriptional plasticity, and rising antimicrobial resistance. However, how these features integrate into coordinated bacterial states within the tumor microenvironment remains unclear. This study integrates virulence, expression, and antimicrobial signatures to define potentially meaningful *H. pylori* profiles.

**Methods:**

We analyzed 159 histologically confirmed, *H. pylori*-positive gastric cancer tissues. Virulence gene presence, expression, and antimicrobial resistance–associated mutations were quantified using polymerase chain reaction–based assays. Twenty-one molecular features were standardized and integrated using unsupervised *k*-means and TwoStep clustering. Associations with clinicopathological characteristics were evaluated using false-discovery-rate-adjusted statistical tests.

**Results:**

Two dominant *H. pylori* molecular states were identified: a virulence-competent Colonizer profile (127/158, 80.4%) enriched for *cagA*/*vacA* gene carriage and expression (*q* < 0.001), and a putative Resistant Inflammatory profile (31/158, 19.6%) characterized by elevated *iceA* expression (*q* = 0.002) and clarithromycin resistance mutations (*q* = 0.022), with reduced *cagA*/*vacA* (*q* < 0.001). Histological detectability of *H. pylori* differed markedly between profiles (91.3% vs 45.2%, *p* < 0.001). Univariate differences were observed in tumor differentiation (*p* = 0.047) and anatomical distribution (*p* = 0.042), while multivariable associations were directional but non-significant. Four-cluster modelling identified additional sub-phenotypes.

**Conclusions:**

Integrated profiling suggests that *H. pylori* adopts distinct molecular states in gastric cancer associated with specific clinicopathological features. These findings support systems-level microbial characterization as a hypothesis-generating framework for exploring whether microbial molecular states may contribute to future risk stratification and eradication strategies in AMR-endemic regions, pending functional and external validation.

## 1 Introduction

*Helicobacter pylori*–associated gastric cancer emerges within a dynamic host–microbe ecosystem shaped by chronic inflammation, immune remodeling, epithelial transformation, and cumulative therapeutic exposure. While individual virulence determinants and antimicrobial resistance (AMR) mechanisms are well documented, data are scarce regarding how these features integrate into coordinated pathogenic profiles within human gastric cancer tissues. Furthermore, it remains unclear how such profiles correlate with specific tumor phenotypes and clinical contexts (Duan et al., 2025; Malfertheiner et al., 2022; Weng et al., 2021).

Recent genomic and transcriptomic studies indicate that *H. pylori* populations exhibit marked heterogeneity, enabling rapid adaptation to distinct gastric microenvironments (Alva et al., 2025). This diversity allows for spatially resolved transcriptional adaptation to microniches, particularly within the complex tumor ecosystem (Xiao and Ma, 2022). Within malignant gastric tissue, bacteria encounter selective pressures that differ fundamentally from those in non-neoplastic mucosa, including hypoxia, altered pH, stromal remodeling, immune surveillance, and prior antimicrobial exposure (Kuo and Kao, 2025; Liu et al., 2025; Backert, 2019). These pressures may shape combinatorial patterns of virulence activity and resistance mutations, giving rise to distinct molecular phenotypes (Yamada et al., 2025; Dawan and Ahn, 2022).

Despite advances in virulence genotyping and resistance surveillance, most prior investigations have examined isolated markers or relied on cultured isolates. This limits our insight into integrated bacterial behavior within the complex tumor microenvironment (Emmanuel et al., 2025; Zhang et al., 2025). Multidimensional, tissue-based studies combining gene presence, transcriptional activity, and resistance profiling remain rare (Tan et al., 2025; Zhang et al., 2024; Kinoshita-Daitoku et al., 2021). Furthermore, the application of unsupervised analytical frameworks to identify emergent bacterial patterns in cancer-associated tissues is currently underexplored (Lai et al., 2025).

To address these limitations, this study performs an exploratory integrated *in vivo* molecular profiling of *H. pylori* within histologically confirmed gastric cancer tissues. We hypothesized that *H. pylori* within the tumor microenvironment may not exist as a collection of independent traits, but as integrated molecular states where antibiotic resistance mutations and virulence expression levels are co-dependent. Specifically, we investigated the potential relationships between the metabolic costs associated with multiple resistance mutations and observed shifts in virulence transcription, which may shape the bacteria’s pathogenic potential. To test this, we combined virulence gene presence, expression, and resistance-associated mutations into a unified analytical framework of 21 standardized markers. In this context, we define ‘molecular profiles’ as the integrated constellation of these 21 standardized markers, which represent hypothesized ‘molecular states’—coordinated bacterial phenotypes characterized by specific trade-offs between virulence and antimicrobial evasion. Using unsupervised clustering, we delineate these putative distinct molecular profiles and examine their associative relationships with tumor anatomical location, histological subtype, differentiation status, and inflammatory context. This approach reframes *H. pylori* heterogeneity as a potentially tumor-contextual and pattern-driven phenomenon, offering a preliminary system-level perspective on microbial adaptation within malignant gastric tissue.

## 2 Methodology

### 2.1 Study design and setting

This study analyzed *H pylori*–positive gastric cancer tissues obtained from patients treated at three major tertiary care hospitals in Islamabad and Rawalpindi, Pakistan: Pakistan Institute of Medical Sciences (PIMS), Holy Family Hospital, and Pak Emirates Military Hospital Rawalpindi. This study integrates virulence gene profiling, transcriptional activity, and resistance-associated mutations to characterize the molecular diversity of *H. pylori* in gastric cancer. We further evaluate how these microbial signatures correlate with host clinicopathological features. All laboratory analyses were performed at the Cancer Genetics and Epigenetics Laboratory, COMSATS University Islamabad (CUI), using de-identified gastric biopsy tissues.

### 2.2 Study population

Gastric biopsy specimens were obtained as part of routine diagnostic evaluation from patients undergoing endoscopic or surgical assessment within the 3 hospitals. All tissues were processed immediately after collection according to standard histopathology protocols for gastric cancer diagnosis. Formalin−fixed paraffin−embedded (FFPE) blocks were prepared, sectioned, and stained with hematoxylin–eosin (H&E) for primary tumor assessment. A consultant gastrointestinal pathologist independently verified each diagnosis, confirming the presence of gastric cancer, assessing tumor differentiation, and determining tumor cell content.

### 2.3 Tissue selection and *H. pylori* diagnosis

A total of 200 gastric biopsy specimens, including tumors with and without invasive edges, were processed as part of routine diagnostic evaluation. *H. pylori* infection was assessed using May–Giemsa staining. Stained sections were examined at 40× magnification using an Olympus BX41 light microscope (Olympus, California, USA).

### 2.4 Eligibility criteria

Patients were eligible for inclusion if they had histologically confirmed gastric cancer, available FFPE tissue blocks with sufficient material for DNA and RNA extraction, and detectable *H. pylori* DNA by PCR. Only specimens with adequate tumor representation (≥80% tumor cells on H&E review) and acceptable nucleic acid integrity were included. Cases were excluded if tissue quantity or quality was insufficient, if severe gastric comorbidities compromised tissue integrity, or if prior neoadjuvant therapy (chemotherapy, radiotherapy, or chemoradiotherapy) had resulted in marked RNA degradation. Patient consent for molecular analyses was documented at the time of clinical evaluation. The final analytic cohort therefore comprised only those samples meeting all histological, molecular, and quality-control requirements.

### 2.5 Clinical cohort selection

To characterize *H. pylori* molecular heterogeneity within the tumor microenvironment, only samples with detectable *H. pylori* DNA were included in downstream analyses. Of the 200 gastric cancer specimens initially processed, 41 (20.5%) showed no amplification of *H. pylori* virulence genes or the internal housekeeping genes (*glmM, hsp60*, and *ureA*) and were therefore excluded. The final analytic cohort comprised 159 *H. pylori*–confirmed cases to support preliminary transcriptional and mutational profiling within this specific geographic context.

### 2.6 Clinical variable harmonization

#### 2.6.1 Tumor node metastasis-based cancer staging

Tumor stage was derived directly from the raw TNM classifications recorded in the clinical dataset. Each case included separate entries for the primary tumor (T), regional lymph nodes (N), and distant metastasis (M). These TNM components were first combined into a unified TNM descriptor (e.g., T2N0M0, T4N1M0, T4N1M1), following standard oncological conventions. Using these composite TNM codes, overall cancer stage (I–IV) was assigned according to internationally accepted gastric cancer staging criteria, as defined in the 8th edition of the American Joint Committee on Cancer (AJCC) Cancer Staging Manual (American Joint Committee on Cancer, 2017).

Once the stage group was determined, cases were further classified into a binary staging variable to support downstream statistical comparisons. Stage I and Stage II tumors were grouped as early-stage disease, reflecting localized or locally advanced tumors without extensive nodal or metastatic spread. Stage III and Stage IV tumors were grouped as late-stage disease, representing regionally advanced or metastatic cancer. This dichotomization ensured adequate statistical power and aligned with clinical decision-making frameworks commonly used in gastric cancer management.

#### 2.6.2 Tumor location

Tumor location was recorded using heterogeneous anatomical descriptors across pathology and clinical reports. These raw entries were harmonized into a four-level variable reflecting clinically meaningful anatomical regions. Distal stomach tumors included those arising in the antrum, pylorus, or distal body. Proximal stomach tumors included those involving the fundus, cardia, or proximal body. Cases describing diffuse, pangastric inflammation, or non-specific gastric involvement were grouped as whole-stomach or non-specific gastric tumors. Tumors with invasion into adjacent organs or extension into the duodenum or esophagus were classified as multi-organ or upper gastrointestinal extension. This harmonization ensured consistent classification across institutions and prevented misinterpretation of diffuse or advanced tumors.

#### 2.6.3 Histological classification

Histological type was harmonized from pathology reports that used varied terminology. All adenocarcinoma subtypes—including intestinal, diffuse, mixed, poorly cohesive, and signet-ring cell carcinomas—were grouped under a single adenocarcinoma category, consistent with WHO classification. Non-adenocarcinoma malignancies, including neuroendocrine tumors, lymphomas, and gastrointestinal stromal tumors, were grouped separately. This distinction reflects the biological and clinical differences between adenocarcinoma and other gastric tumor types.

#### 2.6.4 Differentiation grade

Differentiation grade (G) was standardized from variable pathology descriptors. Well−differentiated tumors included cases with well−formed glandular structures, consistent with G1 adenocarcinoma. Moderately differentiated tumors represented intermediate gland formation G2. Poorly differentiated tumors included G3 adenocarcinomas as well as poorly cohesive and signet−ring cell carcinomas, which are classified as high−grade lesions under WHO criteria (WHO, 2019). Non−malignant or atypical cases—including chronic gastritis, intestinal metaplasia, dysplasia, or atypical but noninvasive lesions were grouped separately. This category was retained because several *H. pylori*–positive cases lacked overt malignancy, which is biologically relevant to interpreting bacterial phenotypes.

#### 2.6.5 Other clinical variables

TNM-derived stage, tumor location, histological type, and differentiation grade were complemented by additional harmonized variables. Socio demographic variables, including age at diagnosis and sex, were extracted from clinical records and harmonized across institutions. Smoking status was recoded as either current/former smoker or never smoker. Survival outcomes were grouped into deceased, under treatment, or cured, reflecting the categories used in the clinical registry.

**Figure 1:**
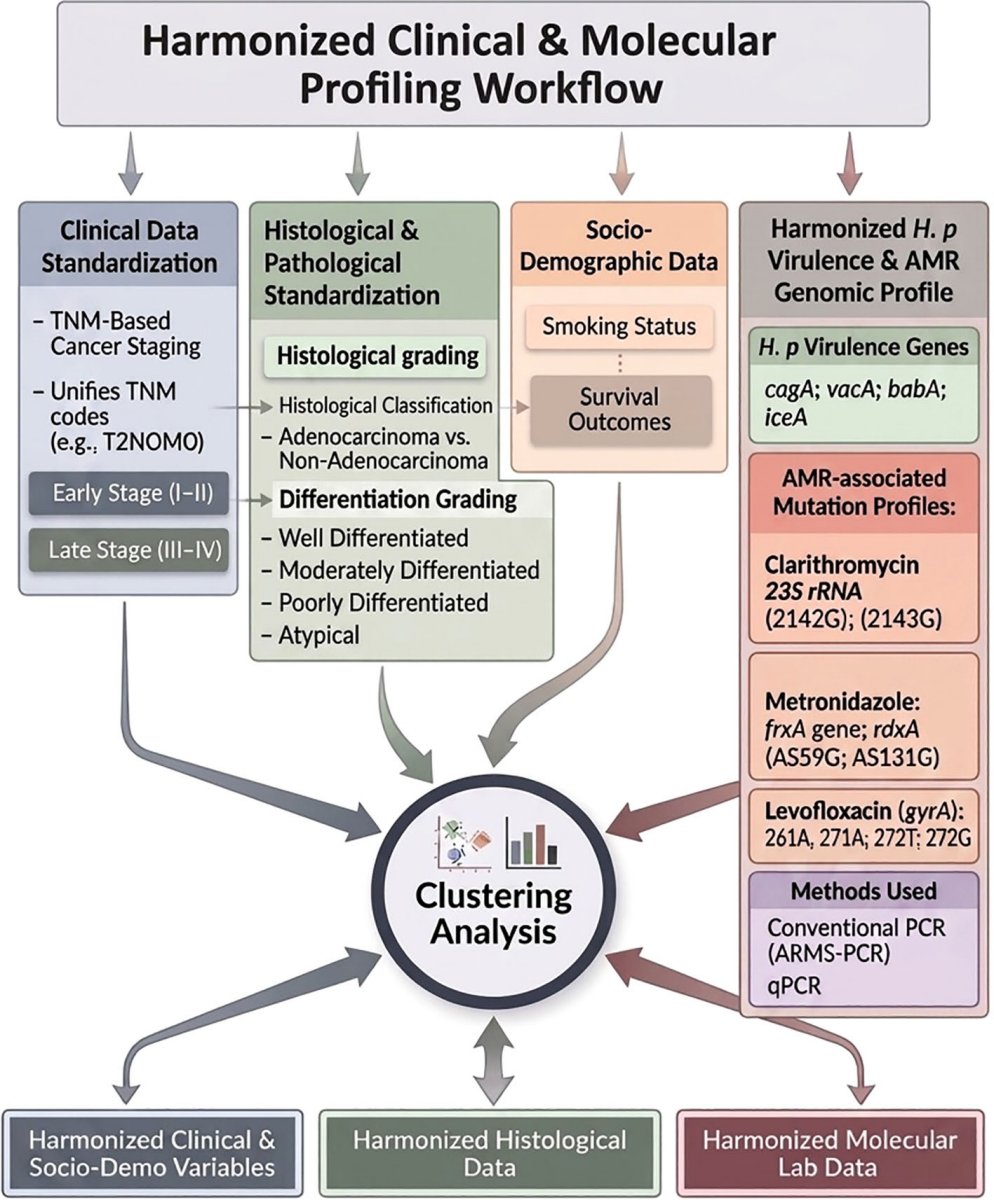
Integrated workflow for clinical harmonization and molecular profiling. Workflow for clinical harmonization, molecular profiling, and integrated clustering of *H. pylori*–associated gastric cancer data. The schema illustrates the integration of harmonized clinical and socio-demographic variables with molecular data, including virulence gene presence (*cagA, vacA, babA, iceA*), gene expression levels, and AMR-associated mutations. Mutation profiles for clarithromycin (*23S rRNA*), metronidazole (*rdxA, frxA*), and levofloxacin (*gyrA*) were standardized and integrated into a unified analytic framework for unsupervised clustering.

This harmonized clinical dataset, paired with high resolution molecular profiling of *H. pylori*, provided a unified framework for examining how bacterial virulence, transcriptional activity, and resistance-associated mutations relate to the pathological context of gastric cancer. An overview of the harmonized clinical, pathological, socio−demographic, and molecular data integration workflow is presented in **Figure 1**.

### 2.7 Integrated molecular detection: virulence expression and mutation genotyping

Total genomic DNA and RNA were isolated from approximately 70 mg of gastric biopsy tissue using a modified TRIzol™ phase-separation protocol. Tissues were homogenized under chilled conditions to preserve transcript integrity. Following phase separation, DNA was recovered from the organic phase and subjected to multiple washes in 0.1 M sodium acetate to remove heme and other PCR inhibitors. Comprehensive details of the dual-phase harvesting and extraction protocol are provided in **Supplementary Section S1.1**.

Nucleic acid purity and integrity were assessed prior to downstream analysis. Samples were considered acceptable for transcriptional profiling only when DNA (A_260_/A_280_) ratios ranged between 1.7 and 1.8, RNA ratios approximated 2.0, and agarose gel electrophoresis confirmed minimal degradation. These quality control criteria ensured reliable downstream amplification. Detailed quality control procedures are described in **Supplementary Section S1.2**.

Complementary DNA (cDNA) was synthesized from total RNA using the SuperScript™ III First-Strand Synthesis System with random hexamers. Standardized reagent composition and thermal cycling parameters for reverse transcription are provided in **Supplementary Tables S3**, **S5**.

Relative expression of *H. pylori* virulence genes (*cagA, vacA, babA*, and *iceA*) was quantified using SYBR™ Green–based real-time PCR on the ABI PRISM 7000/7500 platform. Each reaction was performed using standardized reagent composition and primer concentrations as detailed in **Supplementary Table S4**. Analytical specificity was confirmed by melt-curve analysis for each amplification. Amplifications were performed in triplicate to ensure reproducibility.

*Ct* values were normalized to the geometric mean of three housekeeping genes (*glmM, ureA*, and *hsp60*), which served as internal controls for bacterial load and RNA integrity. To ensure high-fidelity reporting, a threshold of ΔCt > 1.0 was established to define positive transcripts. Markers falling below this limit were considered transcript-negative to prevent inclusion of background noise. Expression values were converted to 2^−ΔCt and categorized into tertiles (low, moderate, high) to ensure comparability with binary resistance markers during clustering and to minimize the impact of extreme transcriptional outliers on the *k-*means algorithm.

Primers used for virulence gene detection and housekeeping gene normalization were custom designed and bioinformatically validated for thermodynamic stability, specificity, and absence of secondary structures. Full primer sequences, amplicon sizes, and melting temperatures are provided in **Supplementary Table S1**.

Antibiotic resistance–associated mutations were detected using allele-specific PCR approaches. Clarithromycin resistance was evaluated using tetra-primer amplification refractory mutation system (tetra-ARMS PCR) targeting A2142G and A2143G mutations in the *23S rRNA* gene. Amplicon patterns distinguished wild-type and mutant alleles within a single reaction. Reaction composition and thermal cycling conditions for tetra-ARMS PCR are provided in **Supplementary Tables S5**, **S6**, respectively.

Metronidazole resistance was assessed through detection of mutations in *rdxA* (codons 59 and 131) and *frxA* genes, while levofloxacin resistance was evaluated by screening *gyrA* mutations at codons 261, 271, and 272. To minimize cross-reactivity and account for primer melting temperature differences, each mutation was amplified in an independent reaction rather than multiplexed.

All resistance genotyping reactions were performed in standardized 16–25 µL volumes using approximately 1 µg genomic DNA template. PCR products were resolved on 2% agarose gels and visualized under UV illumination. Mutation status for each locus was coded as present or absent for downstream clustering analysis. Primer sequences and thermodynamic properties for antimicrobial resistance genotyping are provided in **Supplementary Table S2**.

A complete summary of primer sequences, reagent compositions, and thermal cycling parameters for all transcriptional and resistance assays is provided in **Supplementary Tables S1**–**S6**.

### 2.8 Data integration and harmonization

All clinical, pathological, and molecular variables were merged into a unified analytic dataset prior to statistical evaluation. Continuous molecular features, including virulence gene expression values, were standardized to *z*-scores to ensure equal weighting across variables during clustering. Binary variables, such as virulence gene presence and antibiotic resistance–associated mutations, were encoded as 0/1. One extreme molecular outlier (the *rdxA* AS131G variant, *z* = 12.5) was excluded to prevent distortion of distance-based algorithms. All clinical and molecular variables were complete across the analytic cohort, eliminating the need for imputation procedures. This harmonized dataset formed the foundation for all unsupervised clustering analyses and clinicopathological comparisons.

### 2.9 Clustering and statistical analysis

Unsupervised clustering was performed to characterize molecular heterogeneity among *H. pylori*–positive gastric cancer tissues. We applied *k*-means clustering to 21 standardized virulence and resistance features in R (version 4.3.1). The selection of these 21 molecular markers was based on their established roles in *H. pylori* pathogenesis across three functional axes: (i) canonical virulence factors associated with oncogenic signaling *(cagA, vacA, babA, iceA*), (ii) transcriptional activity as a proxy for *in vivo* fitness and niche adaptation, and (iii) primary AMR mutations in the *23S rRNA*, *rdxA* and *gyrA genes*. This comprehensive panel allowed for characterization of observed bacterial molecular states. While these markers represent key functional axes, the resulting profiles are considered hypothesis-generating and require further functional validation.

The optimal number of clusters (*k* = 2) was determined using the elbow method and hierarchical clustering. As detailed in Section 2.8, one extreme molecular outlier was excluded to prevent distortion of the standardized feature space and ensure the identified profiles remained representative of the broader cohort’s clinical molecular landscape. To ensure equal weighting across diverse biological dimensions (genomic, transcriptomic, and mutation data), all features were integrated via feature-wise *z-score* standardization, preventing numerically dominant variables from biasing the Euclidean distance calculations.

To assess cluster robustness and compare results with alternative manifold learning approaches, we applied a non-linear dimensionality reduction using t-distributed Stochastic Neighbor Embedding (*t-*SNE), calculated average silhouette widths, and conducted bootstrap resampling (*B* = 1,000 iterations) to estimate Jaccard similarity coefficients. While alternative matrix factorization techniques such as Non-negative Matrix Factorization (NMF) were considered, *k*-means was selected to provide discrete, clinically actionable molecular stratifications. Feature importance was quantified as the absolute difference in standardized cluster means, allowing identification of the most discriminating molecular markers. To explore finer−scale intra-cluster heterogeneity, a complementary TwoStep clustering algorithm (using log-likelihood distance and Schwarz’s Bayesian Criterion) yielded four refined molecular sub-profiles nested within the primary two-cluster structure.

Associations between molecular profiles and clinicopathological variables were assessed. Categorical variables were compared using Pearson’s chi-square test or Fisher’s exact tests. Continuous variables were evaluated using the Mann–Whitney U test or Kruskal–Wallis test for non-normally distributed data. Differences in standardized molecular features across clusters were examined using one-way ANOVA followed by *post-hoc* testing where applicable. Variables were selected for the multivariable model based on a univariate screening threshold (*p* < 0.10). Consequently, host-level factors such as age and smoking status were excluded from the final model as they demonstrated no association with cluster assignment in the univariate analysis (*p* > 0.30; **Supplementary Table S7**), allowing for a more parsimonious model focused on exploring tumor-specific characteristics. It is acknowledged that this observational framework identifies associations rather than causal links. To account for multiple comparisons, *p*-values for clinical and molecular associations were adjusted using the Benjamini–Hochberg False Discovery Rate (FDR) method. Statistical significance was defined as an FDR-adjusted *q* < 0.05. Molecular visualizations, including heatmaps, *t-*SNE and principal component analysis (PCA) plots, were generated in R using *ggplot2*, *pheatmap*, and *FactoMineR*.

## 3 Results

### 3.1 Identification of two distinct *H. pylori* molecular profiles in gastric cancer tissues

To characterize molecular heterogeneity among *H. pylori* genotypes infecting gastric cancer tissues, we performed unsupervised clustering of integrated virulence expression patterns and antibiotic resistance–associated genotypes. Virulence gene expression (*cagA, vacA, babA, iceA*) was categorized into tertiles based on 2^-^Δ*Ct* values, and resistance-associated mutations were coded as binary variables. All 21 molecular features were standardized to *z*-scores prior to clustering.

**Figure 2:**
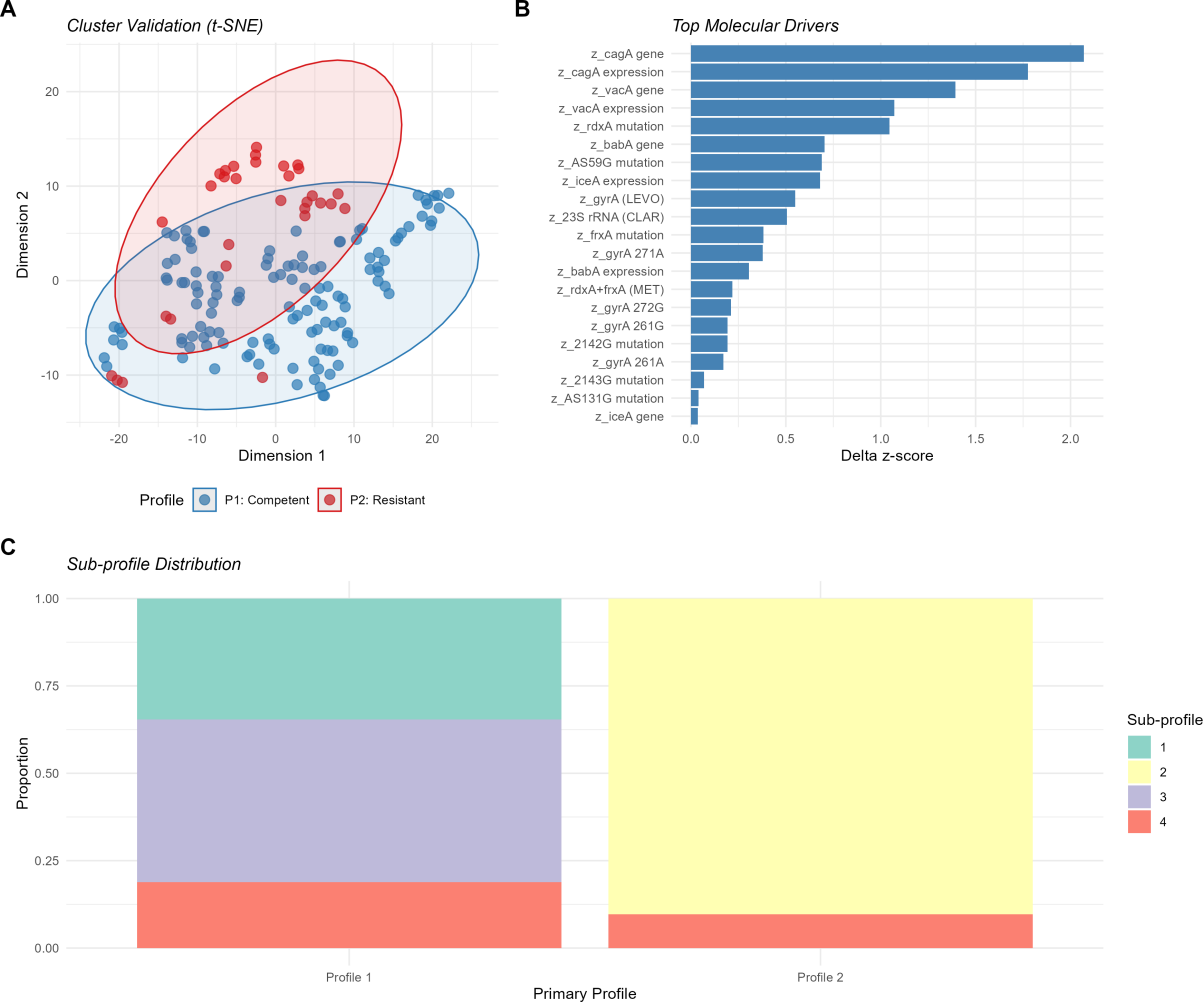
Validation of *H. pylori* molecular heterogeneity and feature importance. **(A)** *t-*SNE plot validating the two-cluster solution identified by *k*-means; ellipses represent 95% CI, demonstrating clear non-linear separation between Profile 1 (Competent Colonizer) and Profile 2 (putative Resistant Inflammatory). **(B)** Feature importance scores based on absolute difference in standardized cluster means (Δ*z-*scores); markers are ranked by their discriminatory power, with *cagA* and *vacA* defining the oncogenic axis, and *iceA* and *23S rRNA* (CLAR) mutations driving the putative resistant-inflammatory phenotype. **(C)** Nested sub-profile distribution showing the stability of the two primary profiles when modeled across finer-scale heterogeneity. All markers labeled with a *z_* prefix indicate standardized scores relative to the cohort mean (n = 158).

Initial *k*-means clustering (*k* = 3) identified one extreme outlier (AS131G mutation, *z* = 12.5), which was excluded from primary analysis (n = 158) to ensure algorithmic stability. Sensitivity analysis confirmed the robustness of the clustering solution, with 98.7% classification consistency observed when the AS131G outlier was included in the model. Hierarchical clustering and elbow method both supported a two-cluster solution, which was further validated by *t-*SNE visualization (**Figure 2A**). Bootstrap validation (n=1,000 iterations) yielded mean Jaccard similarity coefficients of 0.641 for Profile 1 and 0.704 for Profile 2. While these values indicate moderate stability, they suggest that the two identified profiles represent reproducible molecular trends within the cohort.

**Figure 3:**
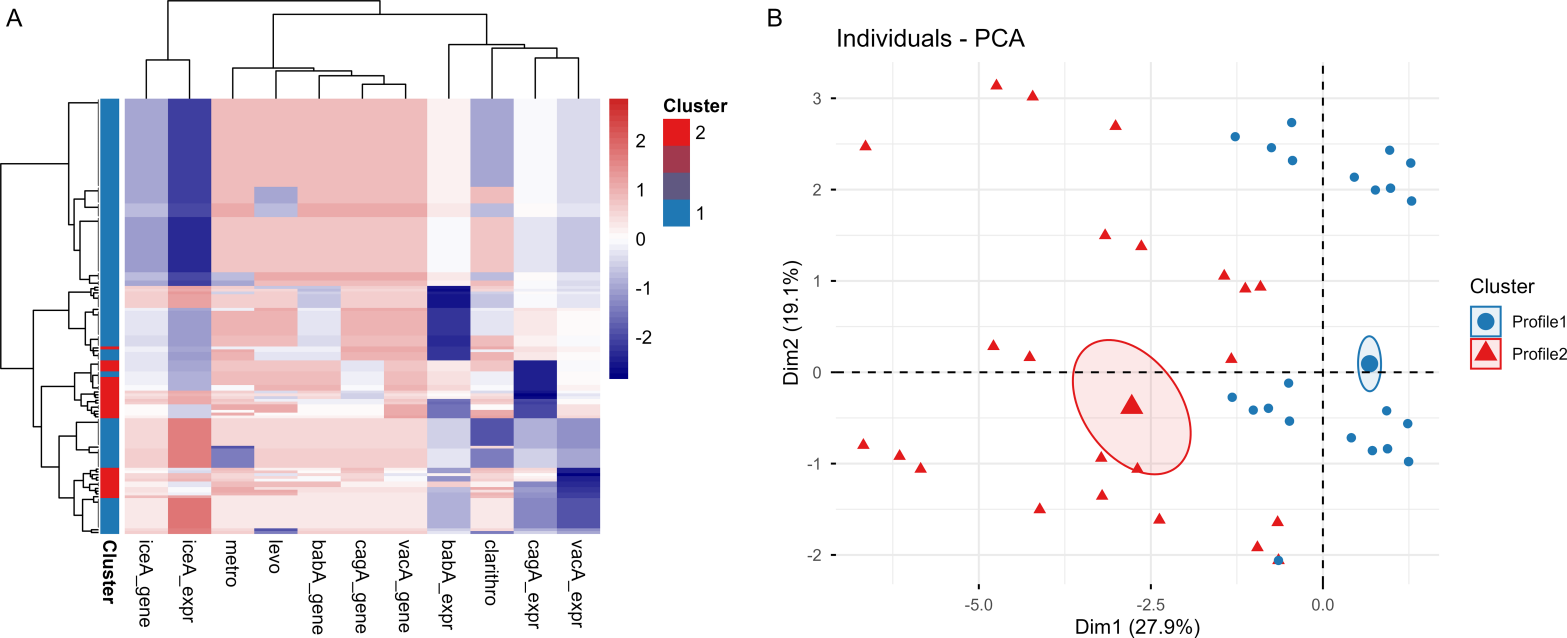
Molecular profiling of *H. pylori* virulence and resistance features in gastric cancer tissues. **(A)** Heatmap of standardized virulence gene presence, virulence gene expression (*z*-scores), and antibiotic resistance class markers across *H. pylori*–positive samples. Rows represent *H. Pylori*-positive samples and columns represent molecular features. Colors indicate relative feature intensity after row-wise scaling. The annotation bar denotes the two molecular profiles identified by *k*-means clustering. **(B)** Principal component analysis of the same feature set showing clear separation between Profile 1 and Profile 2 along the first two principal components. Ellipses represent 95% CI for each cluster.

Two statistically divergent molecular patterns emerged: Profile 1 (n = 127) was characterized by significantly higher standardized scores for *cagA* and *vacA* (gene presence and expression) whereas Profile 2 (n = 31), was characterized by significantly lower virulence factor scores paired with increased scores for specific resistance-associated mutations (**Table 1**; **Figure 3**). Notably, 11 of the 21 features maintained statistical significance after FDR correction, with *cagA* transcriptional signatures and *rdxA* resistance mutations serving as the primary divergent markers between these two putative molecular states.

**Table 1:** Distribution of standardized molecular features by *H. pylori* profile (n = 158).

Feature category	Specific marker	Profile 1 (n = 127)	Profile 2 (n = 31) [95% CI]	Adjusted *p* (*q*)
Virulence Expression	*cagA* expression	0.35	-1.42 [-1.75, -1.09]	**<0.001**
	*vacA* expression	0.21	-0.86 [-1.18, -0.54]	**<0.001**
	*babA* expression	0.05	-0.25 [-0.57, 0.07]	0.193
	*iceA* expression	-0.14	0.54 [0.22, 0.86]	**0.002**
Virulence Presence	*cagA* gene	0.42	-1.65 [-1.98, -1.32]	**<0.001**
	*vacA* gene	0.27	-1.12 [-1.44, -0.80]	**<0.001**
	*babA* gene	0.13	-0.57 [-0.89, -0.25]	**<0.001**
	*iceA* gene	0.00	-0.04 [-0.36, 0.28]	0.857
Clarithromycin	Resistance mutations	-0.09	0.41 [0.09, 0.73]	**0.022**
	2142G mutation	-0.03	0.16 [-0.16, 0.48]	0.395
	2143G mutation	-0.01	0.06 [-0.26, 0.38]	0.772
Metronidazole	Resistance mutations	0.06	-0.16 [-0.48, 0.16]	0.378
	*frxA* mutations	0.08	-0.30 [-0.62, 0.02]	0.095
	*rdxA* mutations	0.21	-0.83 [-1.15, -0.51]	**<0.001**
	AS59G mutation	0.14	-0.55 [-0.87, -0.23]	**<0.01**
	AS131G mutation	-0.07	-0.11 [-0.43, 0.21]	**0.004**
Levofloxacin	Resistance mutations	0.10	-0.45 [-0.77, -0.13]	**0.012**
	*gyrA* 261A	-0.03	0.14 [-0.18, 0.46]	0.438
	*gyrA* 261G	0.04	-0.15 [-0.47, 0.17]	0.395
	*gyrA* 271A	0.07	-0.31 [-0.63, 0.01]	0.059
	*gyrA* 272T	0.03	-0.11 [-0.43, 0.21]	0.389
	*gyrA* 272G	0.04	-0.17 [-0.49, 0.15]	0.389

Values represent standardized *z*-scores (mean = 0, SD = 1). The 95% CI corresponds to the mean *z*-score within Profile 2 (*n* = 31). Adjusted *p*-values (*q*-values) were calculated using the Benjamini–Hochberg FDR procedure to account for multiple testing across the 21 molecular features. Bold values indicate statistical significance (*q* < 0.05).

As illustrated in **Table 1**, Profile 1 was associated with the presence and moderate expression of oncogenic virulence factors including *cagA* (*z* = 0.42) and *vacA* (z = 0.27). The profile also showed near-average expression of adhesion-related *babA* (*z* = 0.05) and below-average inflammatory *iceA* expression (*z* = -0.14). Among resistance markers, Profile 1 exhibited higher prevalence of metronidazole-associated *rdxA* mutations (*z* = 0.21) and AS59G variants (*z* = 0.14) compared to Profile 2.

In contrast, Profile 2 was characterized by elevated inflammatory *iceA* expression (*z* = 0.54, FDR-adjusted *q* = 0.002) as highlighted in the Feature Importance analysis (**Figure 2B**). This profile demonstrated a frequent absence or reduced transcription of canonical virulence genes, including *cagA* (*z* = -1.65) and *vacA* (*z* = -1.12). While individual *gyrA* SNPs showed varying distributions, the overall levofloxacin resistance burden was significantly lower in this profile (*z* = -0.45, FDR-adjusted *q* = 0.012), whereas clarithromycin resistance markers were significantly enriched (*z* = 0.41, FDR-adjusted *q* = 0.022).

Further exploration of the internal structure of these clusters using nested clustering revealed stable sub-groupings within each primary profile (**Figure 2C**), pointing toward finer-scale adaptation. Together, these findings are consistent with two distinct bacterial adaptive strategies within the gastric cancer microenvironment: one pattern suggestive of a putative oncogenic colonizer phenotype with intact virulence machinery, and a second pattern characterized by a treatment-selected putative inflammatory phenotype marked by clarithromycin resistance and reduced carriage of canonical virulence genes. These interpretations remain associative and require further functional validation.

### 3.2 Feature importance: drivers of molecular separation

**Figure 4:**
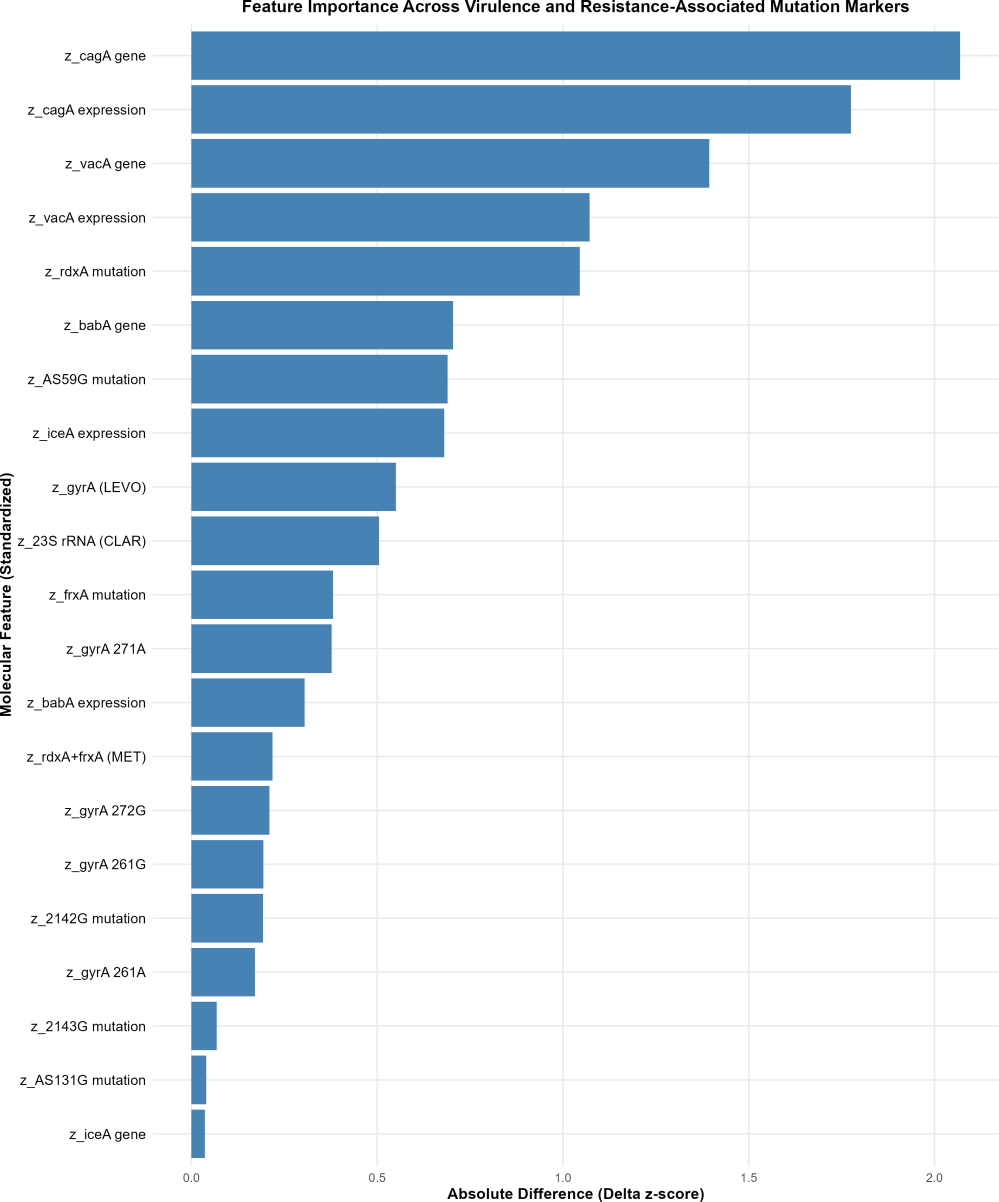
Key molecular drivers of bacterial profile separation. Feature importance across virulence and antibiotic resistance-associated markers. Importance scores reflect the absolute separation between cluster means (*z-*scores) in the *k-*means two-cluster model. The presence and expression of *cagA* and *vacA* emerged as the strongest discriminatory features, defining the high-oncogenic axis of the Competent Colonizer profile, while *iceA* expression and clarithromycin resistance markers characterized the putative Resistant Inflammatory phenotype.

To identify the molecular determinants that most strongly contributed to the separation of the two *H. pylori* profiles, we quantified feature importance based on the absolute difference in standardized cluster means (*z*-scores). **Figure 4** summarizes these importance scores across all virulence and resistance markers, illustrating that virulence-associated features were the dominant drivers of profile separation in this dataset.

The presence and expression of *cagA* showed the strongest discriminatory power, followed by *vacA* gene carriage and expression, collectively defining the putative high−oncogenic axis of the Competent Colonizer profile. Metronidazole-associated *rdxA* and *AS59G* mutations also contributed substantially, whereas *iceA* expression distinguished the putative inflammatory phenotype of Profile 2. Additional moderate discriminators included *babA* gene presence and clarithromycin resistance–associated mutations.

A detailed ranking of the top discriminating features is provided in **Table 2**, highlighting that a small subset of virulence and resistance markers accounts for the majority of molecular divergence observed between profiles.

**Table 2:** Key discriminating features defining *H. pylori* molecular profiles.

Feature	Category	Importance Score	Interpretation
*cagA* gene presence	Virulence gene	2.07	Primary discriminator; positively associated with Profile 1
*cagA* expression	Virulence expression	1.77	High discriminatory power; enriched in Profile 1
*vacA* gene presence	Virulence gene	1.39	Strong discriminator; positively associated with Profile 1
*vacA* expression	Virulence expression	1.07	Discriminatory marker; enriched in Profile 1
*rdxA* mutation	Metronidazole resistance	1.04	Key resistance discriminator; enriched in Profile 1
*iceA* expression	Virulence expression	0.68	Primary discriminator for Profile 2
AS59G mutation	Metronidazole resistance	0.69	Positively associated with Profile 1
*babA* gene presence	Virulence gene	0.70	Moderate discriminator; enriched in Profile 1
Clarithromycin mutations (overall)	Resistance	0.50	Primary resistance discriminator for Profile 2

Importance scores reflect absolute separation between cluster means (z-scores) in the k-means 2-cluster model.

### 3.3 Clinical and pathological associations of the molecular profiles

To determine whether the two molecular profiles corresponded to differences in host or tumor characteristics, we compared demographic, clinical, and pathological variables across profiles. **Table 3** summarizes the observed key statistically relevant clinicopathological differences between profiles, while the complete distribution of demographic and clinical variables is provided in **Supplementary Table S7**.

**Table 3:** Clinical and pathological characteristics by *H. pylori* molecular profile (2-cluster model, n = 158).

Variable	Category	Profile 1	Profile 2	p-value	Interpretation
*H. pylori* histology	Positive	116 (91.3%)	14 (45.2%)	**< 0.001**	Strongest discriminator; histological detectability markedly reduced in Profile 2
	Negative	11 (8.7%)	17 (54.8%)		
Differentiation grade	Well differentiated	34 (26.8%)	9 (29.0%)	**0.047**	Profile 2 enriched for non-malignant / atypical histology
	Moderately differentiated	36 (28.3%)	3 (9.7%)		
	Poorly differentiated	30 (23.6%)	6 (19.4%)		
	Non-malignant / atypical	27 (21.3%)	13 (41.9%)		
Tumor location	Distal stomach	36 (28.3%)	8 (25.8%)	**0.042**	Profile 2 associated with diffuse or non-specific gastric involvement
	Proximal stomach	29 (22.8%)	7 (22.6%)		
	Whole stomach / non-specific	26 (20.5%)	13 (41.9%)		Profile 1 shows more advanced local extension
	Multi-organ / upper GI	36 (28.3%)	3 (9.7%)		
Clinical stage (binary)	Early (I–II)	81 (63.8%)	22 (71.0%)	0.402	No meaningful stage difference
	Late (III–IV)	46 (36.2%)	9 (29.0%)		
Histological type	Adenocarcinoma	82 (64.6%)	23 (74.2%)	0.309	No difference in tumor histology
	Other	45 (35.4%)	8 (25.8%)		
Survival status	Deceased	45 (35.4%)	7 (22.6%)	0.330	No survival difference between profiles
	Under treatment	53 (41.7%)	14 (45.2%)		
	Cured	29 (22.8%)	10 (32.3%)		

Bold values indicate statistically significant associations (p < 0.05).

The strongest difference was observed in *H. pylori* detectability by histology. Profile 1 demonstrated very high microscopic positivity (91.3%), whereas Profile 2 showed substantially reduced detection (45.2%; *p* < 0.001), despite PCR confirmation of *H. pylori* DNA in all cases. This represented the most pronounced discriminator between the profiles at the pathological level.

Differentiation grade also differed significantly (*p* = 0.047). Profile 2 exhibited a higher proportion of non-malignant or atypical tissue (41.9% vs. 21.3%) and fewer moderately differentiated tumors (9.7% vs. 28.3%). Tumor location showed a similar pattern of divergence (*p* = 0.042): Profile 2 was enriched for whole-stomach or non-specific gastric involvement (41.9% vs. 20.5%), whereas Profile 1 more frequently demonstrated multi-organ or upper gastrointestinal extension (28.3% vs. 9.7%). Distal and proximal stomach involvement did not differ meaningfully between groups.

**Figure 5:**
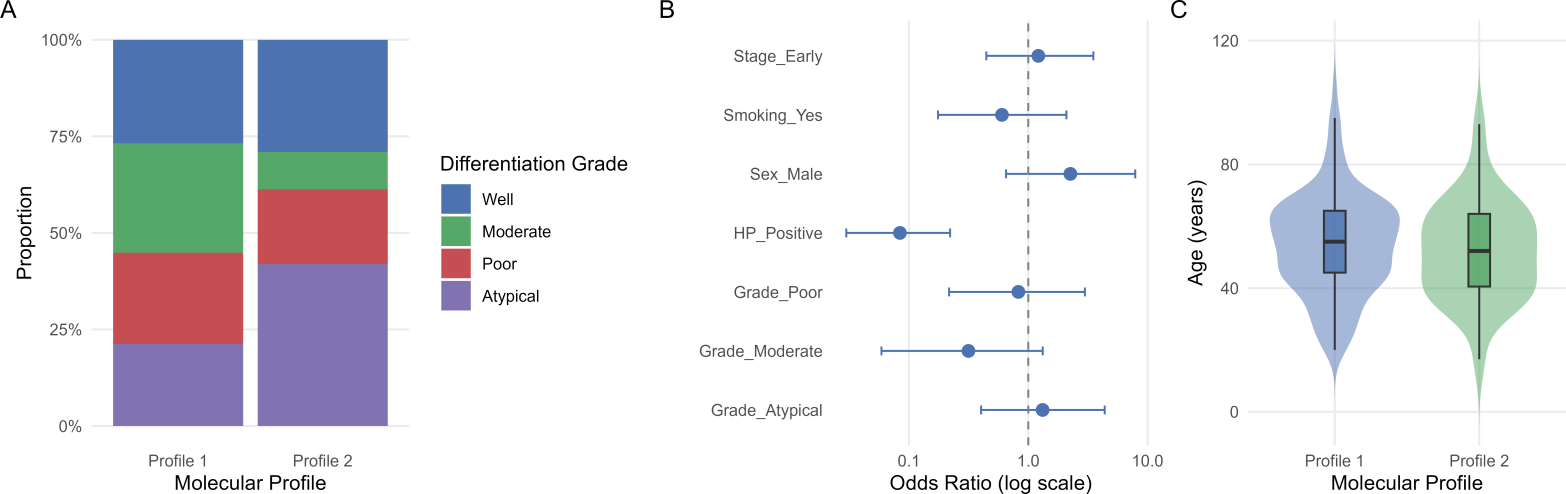
Clinical and pathological associations of molecular profiles. **(A)** Distribution of tumor differentiation grades, showing a higher proportion of poorly differentiated tumors and non-malignant/atypical tissue within Profile 2. **(B)** Multivariable logistic regression identifying clinical predictors of the putative Resistant Inflammatory profile (Profile 2), with odds ratios and 95% CI displayed on a log scale. **(C)** Age distribution across the two molecular profiles.

Demographic variables—including age, sex, smoking status, and year of diagnosis—were comparable between profiles, as were clinical stage, histological tumor type, and survival status (all *p* > 0.30; **Table 3**; **Supplementary Table S7**). To identify independent predictors of the Profile 2 phenotype, a multivariable logistic regression model was constructed (**Figure 5B**). Based on these univariate associations, the model was restricted to tumor-specific variables. Host-level factors, such as age and smoking status, were excluded as they showed no significant association with molecular cluster assignment (*p* = 0.427 and *p* = 0.864, respectively; **Supplementary Table S7**). The model demonstrated directional but non-significant associations for whole-stomach involvement (OR = 1.94; 95% CI: 0.69–5.72) and non-malignant/atypical histology (OR = 1.41; 95% CI: 0.51–4.05) with the Profile 2 molecular state. **Figure 5** illustrates these clinical patterns, showing the distribution of differentiation grades (Panel A), multivariable predictors of Profile 2 (Panel B), the non-significant age distributions (Panel C). Overall, the molecular profiles were similar in demographic characteristics but differed significantly in differentiation grade, tumor location, and the histological detectability of *H. Pylori*.

### 3.4 Summary of molecular and clinical divergence

The integration of molecular and clinicopathological data suggests that the two primary profiles represent divergent bacterial states observed within the gastric cancer microenvironment. Profile 1 is characterized by a higher virulence-to-resistance ratio and high histological detectability, while Profile 2 exhibits a signature of increased antimicrobial resistance and inflammatory expression (*iceA*) paired with reduced microscopic visibility. A consolidated summary of these data-driven characteristics is provided in **Supplementary Table S8**. To determine whether additional molecular substructure existed beyond these two primary axes, we next applied a complementary unsupervised clustering approach. These patterns are presented as associative findings that warrant further functional investigation.

### 3.5 Refinement of molecular profiles using unsupervised twostep clustering

Having established the two primary molecular profiles observed in our cohort, we next explored whether additional substructure existed within these groups. To evaluate finer-scale heterogeneity, we applied an unsupervised TwoStep clustering algorithm using the same 21 molecular features. This complementary approach identified four putative molecular sub-profiles (Clusters 1–4), providing additional granularity and supporting the internal structure of the primary two-cluster model (**Figure 2C**). Clusters 1–2 correspond primarily to subdivisions of the putative Resistant Inflammatory profile, whereas Clusters 3–4 represent subdivisions of the Competent Colonizer profile.

The refined profiles demonstrated significant divergence in virulence signatures and resistance patterns (**Supplementary Table S9**). Cluster 3 (35.2%) was characterized by a putative ‘Metronidazole-Resistant Colonizer’ phenotype, with high *cagA* and *vacA* expression alongside frequent *rdxA* mutations. Cluster 4 (28.3%) represented a hypothesized ‘Multi-Resistant Oncogenic’ profile, demonstrating a high prevalence of clarithromycin resistance markers while maintaining universal *cagA* positivity. Cluster 1 (19.5%) was defined as ‘Low-Virulence Inflammatory,’ showing minimal *cagA/vacA* carriage and elevated *iceA* expression, while Cluster 2 (17.0%) identified a ‘Treatment-Sensitive Oncogenic’ group with high virulence but a low resistance burden. These nested subdivisions suggest that the primary two-cluster structure effectively captures the dominant molecular variations observed within the gastric cancer microenvironment (**Figure 2C**).

### 3.6 Virulence and resistance marker distribution across refined profiles

The distribution of individual virulence and resistance markers further defined the four molecular sub-profiles (**Supplementary Table S10**). Cluster 3 demonstrated the highest prevalence of *vacA* positivity (37.8%) and high *cagA* expression (56.1%). In contrast, Cluster 1 showed the highest proportion of high *iceA* expression (33.9%) but minimal *cagA* presence (3.8%).

Resistance patterns were profile-specific. Clarithromycin resistance markers (A2142G and A2143G) were concentrated in Cluster 4, with 64.3–71.9%, and nearly absent in Cluster 3 (0–1.4%). Metronidazole resistance–associated mutations (*rdxA* gene) were most frequent in Clusters 3 and 4, while Cluster 2 showed the lowest resistance burden, including absence of *rdxA* mutations.

### 3.7 Clinical correlates of the refined four-cluster model

We next examined whether the refined molecular subprofiles corresponded to differences in demographic or clinicopathological characteristics. Detailed clinical associations for the four refined molecular profiles are provided in **Supplementary Table S11**. Demographic variables—including age, sex, and smoking status—did not differ significantly across profiles.

Tumor characteristics showed variable but non-significant trends. Clinical stage demonstrated a non-significant association with cluster membership (p = 0.064), and tumor location varied across profiles without reaching statistical significance (*p* = 0.096). Differentiation grade differed significantly across the four profiles in univariate analysis (*p* = 0.031). Notably, histological detection of *H. pylori* remained the strongest clinical correlate (*p* < 0.001), consistent with the primary model.

### 3.8 Integrative summary across both clustering models

Together, the two-cluster model defined the primary axes of *H. pylori* molecular variation observed in this study. The four-cluster solution provided additional resolution, revealing distinct combinations of virulence and resistance markers with associated clinicopathological patterns. Across both models, significant differences were observed in tumor differentiation and histological detectability (*p* < 0.05). These findings suggest reproducible patterns of bacterial heterogeneity within this cohort, though their biological mechanisms and clinical utility remain subjects for future functional validation.

## 4 Discussion

### 4.1 Principal findings

Our findings suggest that *H. pylori* heterogeneity in gastric cancer follows two distinct associative molecular patterns. Profile 1 (“Competent Colonizer”) reflects a putative oncogenic phenotype characterized by intact *cagA/vacA* machinery and robust transcriptional activity. In contrast, Profile 2 (“putative Resistant Inflammatory”) represents an AMR-associated phenotype defined by clarithromycin resistance and elevated *iceA* expression. This niche-adapted transcriptional state in Profile 2, potentially influenced by antibiotic exposure, may reflect altered bacterial fitness or metabolic activity, which may contribute to the markedly lower histological detectability observed, although this interpretation remains speculative.

Notably, these profiles remained consistent regardless of host smoking status (*p* = 0.864), suggesting decoupling of lifestyle risk factors from the established microbial-tumor niche (Hatakeyama, 2014). This is consistent with large-scale clinico-molecular studies where traditional risk factors do not necessarily dictate specific molecular phenotypes once malignancy is established (The Cancer Genome Atlas Research Network, 2014). Such findings support a model where the localized malignant environment—characterized by unique metabolic and immune stressors—acts as the dominant selective pressure, driving coordinated molecular programs rather than isolated genetic traits.

### 4.2 Tumor microenvironment as a selective niche

The observed clustering of bacterial molecular features suggests that gastric cancer tissue functions as a specialized ecological niche. Compared with non-neoplastic gastric environments, malignant tissue imposes distinct metabolic, immune, and structural constraints, including hypoxia, and epithelial remodeling (Alissa, 2025; Zhang et al., 2023). Mechanistically, these environmental stressors may influence bacterial regulatory programs that favor persistence over rapid replication. However, this interpretation is inferential and based on associative clustering patterns rather than direct functional measurement (Kuo and Kao, 2025). For instance, the transition toward the Profile 2 state may involve the modulation of stress-response regulators that alter cell-wall permeability, potentially linking antibiotic resistance to reduced metabolic activity. Our findings suggest that bacterial evolution in cancer reflects coordinated regulatory programs, rather than random accumulation of independent genetic traits. Such selective pressures may be associated with persistent colonization and prolonged inflammatory signaling, potentially amplifying carcinogenic trajectories.

### 4.3 Expression-level stratification and pathogenic signaling

A major contribution of this work is the demonstration that virulence gene expression adds resolution beyond genotype alone. While prior studies have focused on gene carriage as a predictor of pathogenicity (Maciel et al., 2025), our data indicate that transcriptional activity provides a more granular reflect of the *in vivo* disease state. Unlike the binary classification typical of genotype-only studies, our transcriptional profiling reveals molecular signatures with identical virulence genes can exist in vastly different functional states. This suggests that transcriptional plasticity is a key, yet under-reported, driver of inter-tumor variability.

High *cagA* and *vacA* expression in Profile 1 may be associated with activation of host oncogenic pathways, including NF-κB and STAT3 signaling axes, which are critical for epithelial-to-mesenchymal transition (EMT) and stemness in gastric cells (Ansari and Ahmed, 2025; Salem, 2026). Furthermore, these bacterial states mirror broader molecular mechanisms of host inflammation; for instance, the regulation of systemic cytokines like IL-6 has been linked to microRNA modulators such as miR-203a-3p in other inflammatory pathologies (Jaber et al., 2024), suggesting a conserved role for such molecular axes in mediating host–microbe interactions. This supports expression-based stratification as a functionally meaningful approach, as transcriptional plasticity allows *H. pylori* to tune its inflammatory output in response to the shifting tumor microenvironment.

### 4.4 AMR as a modifier of pathogenic state

Critically, AMR-associated mutations co-segregated with specific virulence expression profiles, suggesting that resistance influences bacterial fitness beyond therapeutic failure. The enrichment of clarithromycin resistance in the low-virulence Profile 2 suggests a potential biological “trade-off.” While this co-segregation is clear, our biological interpretation remains speculative and requires functional validation. Our findings align with ecological models suggesting that resistance-associated mutations—specifically in the *23S rRNA*— have been proposed to impair ribosomal translational efficiency (Andersson and Hughes, 2010; Fuzi, 2025). In the nutrient-limited and high-stress environment of the gastric tumor, the metabolic cost of maintaining such resistance may necessitate the down-regulation of energetically demanding virulence systems, such as the cag Type IV Secretion System (cag-T4SS). We hypothesize that this represents a survival strategy, though functional multi-omic studies are needed to confirm this mechanism.

However, our analysis also revealed important null findings. Individual mutations, such as isolated *gyrA* or specific *23S rRNA* variants, did not consistently correlate with virulence down-regulation, suggesting that any putative “fitness cost” likely results from the integrated molecular state rather than a single genetic switch. These results highlight the complexity of bacterial adaptation and the importance of analyzing multiple markers in combination. Similar patterns of putative reduced fitness have been noted in other AMR-endemic regions (Vieni et al., 2025), reinforcing the view that resistance may actively remodel bacterial physiology in ways that influence host-interaction dynamics and, potentially, the carcinogenic trajectory.

### 4.5 Clinical and translational implications

The association between bacterial molecular profiles and tumor characteristics suggests that *H. pylori* heterogeneity may potentially contribute to inter-tumor variability. Our results align with emerging evidence that *H. pylori*–driven inflammation can modulate immune checkpoint pathways. Specifically, CagA-mediated activation of the STAT3 signaling axis has been shown to up-regulate PD-L1 expression on gastric epithelial cells, suggesting a mechanism for facilitating immune evasion within the tumor microenvironment (He et al., 2025; Ansari and Ahmed, 2025).

Because these microbial states appear to operate independently of traditional host risk factors, as noted in our primary findings, they may represent a distinct layer of information for microbe-informed gastric cancer stratification. While our study is observational and lacks functional immune-profiling data, these patterns support further exploration of bacterial subtypes in risk assessment. These findings highlight potential future avenues for research into precision eradication strategies tailored to bacterial subtypes (Lee, 2026). However, any transition toward clinical decision-making or precision eradication will require extensive prospective validation to determine the physiological impact of these associative molecular patterns.

### 4.6 Methodological strengths and study limitations

Key strengths include the use of histologically confirmed gastric cancer tissues, integration of genomic and transcriptional bacterial data, enabling a multidimensional characterization of *H. pylori* molecular states within the tumor microenvironment. This combined approach allowed for the preliminary assessment of both virulence architecture and antimicrobial resistance–associated adaptations in clinically relevant specimens.

However, several limitations warrant explicit consideration. First, the cross-sectional design precludes definitive causal inferences regarding the temporal relationship between bacterial states and tumor progression. Second, while we identified clear molecular profiles, certain individual markers did not reach statistical significance. For instance, the lack of association with smoking status (*p* = 0.864) and age (*p* = 0.427) suggests that in this cohort, the microbial molecular state may be more heavily influenced by the localized tumor niche than by distal lifestyle factors, as discussed in Section 4.1.

Crucially, the absence of functional assays such as host cytokine profiling or *in vitro* infection models means that the physiological impact of the identified bacterial signatures remains strictly associative. Interpretation of these findings should also consider the geographic context of the Pakistani cohort, which may reflect region-specific antibiotic prescribing patterns and distinct bacterial phylogroups such as *hpAsia2*. Additionally, the analysis was restricted to *H. pylori*-positive tissues to evaluate strain-associated genetic variation inferred directly from tissue-derived DNA. While this allowed for a targeted multivariable analysis of independent predictors (**Figure 5B**), it introduces selection bias and ensures our findings may not generalize to *H. pylori*-negative gastric cancers or early-stage pre-neoplastic lesions.

Finally, the marker focused on canonical virulence and resistance determinants. Emerging genomic studies have identified variation in outer membrane proteins, including *hopL*, as potentially associated with gastric cancer–related strain diversity (Watanabe et al., 2021). However, these observations remain exploratory and lack functional and stage-resolved validation. Future studies integrating expanded genomic loci with functional and spatial transcriptomics approaches will be required to define the micro-spatial heterogeneity of bacterial adaptation and move beyond the correlative patterns reported here.

### 4.7 Conceptual implications and conclusions

In summary, this study advances a multidimensional *in vivo* framework for characterizing *H. pylori* in gastric cancer, suggesting that bacterial populations adopt distinct molecular states shaped by tumor context. By associating integrated microbial molecular signatures with clinicopathological phenotypes, our findings support a shift from single-factor virulence models toward systems-level microbial phenotyping.

These findings support further investigation into whether microbial molecular signatures may complement existing gastric cancer classification frameworks. This work provides a preliminary, hypothesis-generating framework that could eventually inform microbe-guided risk assessment and precision eradication therapy, pending extensive functional and prospective validation.

## Data Availability

Due to ethical restrictions involving human clinical samples and associated molecular data, the datasets generated and analyzed during this study are not publicly available. De‑identified data supporting the findings of this study are available from the corresponding author upon reasonable request and subject to institutional ethical approval. All primer sequences and reference accession numbers used for assay design are provided in the **Supplementary Material**.
