# Supplementary material for: Integrated virulence–resistance profiling of Helicobacter pylori reveals context-dependent pathogenic signatures in gastric cancer
Source: Front Cell Infect Microbiol. 2026 May 19;16:1812371. doi: 10.3389/fcimb.2026.1812371 (PMC13226509; doi:10.3389/fcimb.2026.1812371)
Supplement: Supplementary file 1 [file Table1.docx]

**Supplementary Materials**

**Section S1. Standardized Molecular Protocols**

**S1.1 Dual-Phase Nucleic Acid Extraction**

Total genomic DNA and RNA were isolated from approximately 70 mg of thinly sectioned gastric biopsy tissue using a modified TRIzol™ (Sigma-Aldrich) phase-separation protocol. Tissues were homogenized in 1 mL TRIzol on ice to preserve transcript integrity. Following the addition of 200 µL chloroform and centrifugation (14,000 rpm, 15 min, 4°C), the upper aqueous phase was collected for RNA precipitation using isopropanol. DNA was subsequently recovered from the interphase/organic phase via ethanol precipitation, followed by three washes in 0.1 M sodium acetate to remove heme and other PCR inhibitors commonly present in gastric tissue.

**S1.2 Stringent Quality Control (QC)**

Nucleic acid purity and concentration were determined using the NanoDrop-1000 (Thermo Scientific). Inclusion for transcriptional analysis was gated using the following criteria:

- Genomic DNA: A_260_/A_280_ ratio of 1.7–1.8
- Total RNA: A_260_/A_280_ ratio of approximately 2.0
- Integrity: Verified by 1% agarose gel electrophoresis; samples showing significant degradation or ribosomal subunit smearing were excluded and re-extracted

**S1.3 cDNA Synthesis**

Complementary DNA (cDNA) synthesis was performed using the SuperScript™ III First-Strand Synthesis System (Invitrogen) or RevertAid™ equivalent to generate stable complementary DNA from total RNA. Reaction composition is provided in **Supplementary Table S3**. Reverse transcription thermal conditions are detailed in **Supplementary Table S5**.

**S1.4 RT-qPCR and Normalization Strategy**

Quantitative PCR (qPCR) was performed using SYBR™ Green chemistry on the ABI PRISM 7000/7500 platform. Relative expression was calculated using the 2^−ΔΔCt method and normalized against the geometric mean of three endogenous reference genes: *ureA, glmM*, and *hsp60*. Reaction composition for qPCR is provided in **Supplementary Table S4**. Amplifications were performed in triplicate, and analytical specificity was confirmed using melt-curve analysis.

**S1.5 In Silico Primer Design and Bioinformatic Validation**

Primer design and validation for *H. pylori* virulence markers and housekeeping genes followed a standardized four-step bioinformatic pipeline:

- ***Sequence retrieval:*** Reference sequences were obtained from NCBI GenBank (accessions: AY737319.1, AB017923.1, AF479027.1, AL445067, DQ674277.1)
- ***Primer generation:*** Primers were designed using NCBI Primer-BLAST (taxid:210), prioritizing annealing temperature (T_a_) of 60°C and GC content of 40–60%.
- ***Physicochemical evaluation***: Primers were validated using PCR Primer Stats and Sequence Manipulation Suite (SMS) to confirm absence of secondary structures.
- ***Synthesis:*** Final primers were synthesized by Macrogen Inc. and analyzed using StepOne™ v2.0 software.

**Section S2. Antimicrobial-Associated Resistance (AMR) Genotyping**

**S2.1 Tetra-ARMS PCR for 23S rRNA (Clarithromycin-associated Mutations)**

A tetra-primer amplification refractory mutation system (tetra-ARMS) PCR was optimized to detect A2142G and A2143G mutations. This single-tube reaction utilized two outer primers (FP-1 and RP-1) and two inner allele-specific primers (Fp2143G and Rp2142G). Reaction composition and thermal cycling conditions are provided in **Supplementary Tables S6** and **S5**, respectively.

- Wild-type (wt): 320 bp amplicon (FP-1/RP-1)
- A2142G mutation: 238 bp amplicon (FP-1/RP2142G)
- A2143G mutation: 118 bp amplicon (FP2143G/RP-1)

**S2.2 Allele-Specific PCR Optimization (Metronidazole and Levofloxacin)**

To minimize cross-reactivity and account for melting temperature (T_m_) variations, mutations in *rdxA, frxA,* and *gyrA* were detected in independent reactions. The optimized 25 µL reaction mixture contained 1 µg DNA template, 1.0 µM primers, 12.5 µL master mix, 0.2 mM dNTPs, and 1.5 mM MgCl₂. Amplicons were resolved using 2% agarose gel electrophoresis.

**Section 3. Primer Sequences**

**Supplementary Table S1. qPCR Primers for H. pylori Virulence and Housekeeping Genes**

| **Gene** | **Primer** | **Sequence (5′–3′)** | **Product size (bp)** | **Tm / Annealing (°C)** | **Notes** |
| --- | --- | --- | --- | --- | --- |
| *cagA* | HP-F | AACAGGCGATTTCAGTAAGG | 134 | 60 | Forward |
| *cagA* | HP-R | ACAACCCATTACCGACTAGG | 134 | 60 | Reverse |
| *vacA* | HP-F | AGCATCACACCGCAACAAAG | 187 | 60 | Forward |
| *vacA* | HP-R | CTGCTTGAATGCGCCAAACT | 187 | 60 | Reverse |
| *babA* | HP-F | AGCCGTGCTTTTAGCGATCA | 94 | 60 | Virulence |
| *babA* | HP-R | GCTCTGTTGACCATTGGCAT | 94 | 60 | Virulence |
| *iceA* | HP-F | GCCATGCGGGCTATTTTGAG | 183 | 62 | Virulence |
| *iceA* | HP-R | CCTGGAACACCTAGCTGCAA | 183 | 62 | Virulence |
| *ureA* | HP-F | CAAGTTGATGCTCCACTACGC | 88 | 60 | Housekeeping |
| *ureA* | HP-R | CAAACGTACCGCTTCCACAT | 88 | 60 | Housekeeping |
| *hsp60* | HP-F | CGGGTTGATGACGCATTGAG | 223 | 59 | Housekeeping |
| *hsp60* | HP-R | CGCCATCATAACCGGCATTG | 223 | 59 | Housekeeping |
| *glmM* | HP-F | AAACGCCTTAGTGAGTGCTT | 108 | 61 | Housekeeping |
| *glmM* | HP-R | GCATCACAGCGCATGTCTTC | 108 | 61 | Housekeeping |

**Supplementary Table S2. PCR / ARMS Primers for Antibiotic Resistance–Associated Genes/SNPs**

| Gene | Primer | Sequence (5′–3′) | Product size (bp) | Mutation / SNP | Notes |
| --- | --- | --- | --- | --- | --- |
| *frxA* | HP-F | GGATATGGCAGCCGTTTATCATT | 710 | Not Specific | Conventional PCR |
| *frxA* | HP-R | GAATAGGCATCATTTAAGAGATTA | 710 | Not Specific | Conventional PCR |
| *23S rRNA* | FP-1 | TCGAAGGTTAAGAGGATGCGTCAGTC | 320 | Outer | Tetra-ARMS |
| *23S rRNA* | RP-1 | GACTCCATAAGAGCCAAAGCCCTTAC | 320 | Outer | Tetra-ARMS |
| *23S rRNA* | RP2142G | AGTAAAGGTCCACGGGGTATTCC | 238 | A2142G | Tetra-ARMS |
| *23S rRNA* | FP2143G | CCGCGGCAAGACAGAGA | 118 | A2143G | Tetra-ARMS |
| *gyrA* | 259-F | CACCCCCATGGCGGTT | 280 | Codon 259 | ARMS-PCR |
| *gyrA* | 261G-F | CCCCATGGCGATACG | 280 | Codon 261G | ARMS-PCR |
| *gyrA* | 261A-F | CCCCATGGCGATACA | 280 | Codon 261A | ARMS-PCR |
| *gyrA* | 271A-F | CATGGCGATAATGCGGTTTGTA | 320 | Codon 271A | ARMS-PCR |
| *gyrA* | 271T-F | CATGGCGATAATGCGGTTTGTT | 320 | Codon 271T | ARMS-PCR |
| *gyrA* | 272G-F | ACCCCCATGGCGATAATGCGGTTTATCG | 330 | Codon 272G | ARMS-PCR |
| *gyrA* | HP-F-R | ACTCGCCTTAGTCATTC | — | Common reverse | ARMS-PCR |
| *rdxA* | HP-Fo | GCAGGAGCATCAGATAGTTCT | 886 | Outer | ARMS-PCR |
| *rdxA* | HP-Ro | GGGATTTTATTGTATGCTACAA | 886 | Outer | ARMS-PCR |
| *rdxA* | S59-F | GCATTTTGTGATGGTTACTG | 681 | A59 | ARMS-PCR |
| *rdxA* | AS131-F | TCAACCACAGCATGCAAAG | 463 | R131 | ARMS-PCR |

**Section S4. Reaction Mixtures**

**Supplementary Table S3. Reagents and Volumes for cDNA Synthesis**

| **Reagent** | **Volume (µL)** |
| --- | --- |
| RNA | 1 |
| Random hexamer primer | 1 |
| 5X reaction buffer | 4 |
| RNA inhibitor | 1 |
| DNTPs | 2 |
| Reverse transcriptase | 1 |
| Nuclease-free water | 10 |
| Total volume | 20 |

**Supplementary Table S4. qPCR Reaction Composition (SYBR Green)**

| **Reagent** | **Volume per 11 µL reaction** |
| --- | --- |
| Cdna | 1 µL |
| RNAse-free water | 4 µL |
| 2X SYBR Green master mix | 4 µL |
| Forward primer | 1 µL |
| Reverse primer | 1 µL |
| Total | 11 µL |

**Supplementary Table S5. Thermal Cycling Profiles**

| **Step** | **cDNA Synthesis** | **Tetra-ARMS PCR** |
| --- | --- | --- |
| Initial step | 25°C (5 min), 42°C (60 min) | 95°C (5 min) |
| Cycling (35 cycles) | — | 95°C (15 s), 60.5°C (20 s), 68°C (30 s) |
| Final extension | 80°C (5 min) | 68°C (2 min) |

**Supplementary Table S6. Tetra-ARMS PCR Reaction Mixture (16 µL)**

| **Reagent** | **Volume (µL)** |
| --- | --- |
| DNA template | 1.0 |
| PCR Master Mix | 6.0 |
| PCR water | 3.0 |
| Forward outer primer | 1.5 |
| Reverse outer primer | 1.5 |
| Forward inner primer | 1.5 |
| Reverse inner primer | 1.5 |
| Total | 16.0 |

**Section S5. Results**

**Supplementary Table S7. Clinical and Pathological Characteristics by** *H. pylori* **Molecular Profile (2-Cluster Model, n = 158)**

| **Variable** | **Category** | **Cluster 1 (n = 127)** | **Cluster 2 (n = 31)** | **p-value** |
| --- | --- | --- | --- | --- |
| **Age (years)** | Median (IQR) | 55 (45–65) | 52 (40–65) | 0.427 |
| **Year of diagnosis** | Median (IQR) | 3 (2–4) | 3 (2–4) | 0.695 |
| **Sex** | Male | 68 (53.5%) | 19 (61.3%) | 0.437 |
|  | Female | 59 (46.5%) | 12 (38.7%) |  |
| **Smoking** | No | 84 (66.1%) | 20 (64.5%) | 0.864 |
|  | Yes | 43 (33.9%) | 11 (35.5%) |  |
| **Clinical stage (binary)** | Early (I–II) | 81 (63.8%) | 22 (71.0%) | 0.402 |
|  | Late (III–IV) | 46 (36.2%) | 9 (29.0%) |  |
| **TNM stage (4-level)** | Stage 1 | 35 (27.6%) | 14 (45.2%) | 0.249 |
|  | Stage 2 | 46 (36.2%) | 8 (25.8%) |  |
|  | Stage 3 | 16 (12.6%) | 2 (6.5%) |  |
|  | Stage 4 | 30 (23.6%) | 7 (22.6%) |  |
| **Differentiation grade** | Well differentiated | 34 (26.8%) | 9 (29.0%) | **0.047** |
|  | Moderately differentiated | 36 (28.3%) | 3 (9.7%) |  |
|  | Poorly differentiated | 30 (23.6%) | 6 (19.4%) |  |
|  | Non-malignant / atypical | 27 (21.3%) | 13 (41.9%) |  |
| **Histological type** | Adenocarcinoma | 82 (64.6%) | 23 (74.2%) | 0.309 |
|  | Other | 45 (35.4%) | 8 (25.8%) |  |
| **Tumor location** | Distal stomach | 36 (28.3%) | 8 (25.8%) | **0.042** |
|  | Proximal stomach | 29 (22.8%) | 7 (22.6%) |  |
|  | Whole stomach / non-specific | 26 (20.5%) | 13 (41.9%) |  |
|  | Multi-organ / upper GI | 36 (28.3%) | 3 (9.7%) |  |
| *H. pylori* **(histology)** | Positive | 116 (91.3%) | 14 (45.2%) | **< 0.001** |
|  | Negative | 11 (8.7%) | 17 (54.8%) |  |
| **Survival status** | Deceased | 45 (35.4%) | 7 (22.6%) | 0.330 |
|  | Under treatment | 53 (41.7%) | 14 (45.2%) |  |
|  | Cured | 29 (22.8%) | 10 (32.3%) |  |

**Supplementary Table S8: Biological and Clinical Implications of** *H. pylori* **Profiles**

| **Aspect** | **Profile 1: Competent Colonizer** | **Profile 2: Resistant Inflammatory** |
| --- | --- | --- |
| Virulence strategy | Oncogenic (*cagA*⁺ / *vacA*⁺) | Inflammatory (*iceA*-high) |
| Expression pattern | Moderate virulence expression | High inflammatory, low oncogenic |
| Resistance profile | Metronidazole-associated mutations | Clarithromycin-associated mutations |
| Hypothesized origin | Classic gastric carcinogenesis | Treatment-selected adaptation |
| Clinical concern | Cancer progression | Treatment failure, persistence |

**Supplementary Table S9. Key characteristics of** *H. pylori* **molecular profiles identified by unsupervised clustering in gastric cancer tissues (n = 158)**

| **Profile** | **n (%)** | **Designation** | **Dominant Virulence Pattern** | **Dominant Resistance Pattern** | **Putative Clinical Implication** |
| --- | --- | --- | --- | --- | --- |
| 1 | 31 (19.5%) | Low-Virulence Inflammatory | *cagA*‑ / *vacA*‑, *iceA*‑high | Mixed, moderate clarithromycin | Inflammatory without oncogenic drive |
| 2 | 27 (17.0%) | Treatment-Sensitive Oncogenic | *cagA*+ / *vacA*+, *iceA*+ | Very low resistance | Classic oncogenic, likely treatment‑naïve |
| 3 | 55 (35.2%) | Metronidazole‑Resistant Colonizer | *cagA*‑high / *vacA*‑high | Metronidazole‑high, clarithromycin‑low | Adapted to metronidazole, maintains oncogenicity |
| 4 | 45 (28.3%) | Multi‑Resistant Oncogenic | *cagA*+ / *vacA*+ | Clarithromycin‑high, multi‑resistant | Treatment‑selected oncogenic with broad resistance |

**Supplementary Table S10. Complete Distribution of Variables Across** *H. pylori* **Molecular Clusters (n = 158)**

| **Variable** | **Category** | **Cluster 1 (n = 31)** | **Cluster 2 (n = 27)** | **Cluster 3 (n = 55)** | **Cluster 4 (n = 45)** |
| --- | --- | --- | --- | --- | --- |
| **Virulence genes** |  |  |  |  |  |
| *cagA* | Positive | 1 (3.8%) | 6 (20.6%) | 23 (41.8%) | 15 (34.4%) |
| *vacA* | Positive | 4 (14.2%) | 5 (18.2%) | 21 (38.2%) | 13 (29.7%) |
| *babA* | Positive | 4 (13.7%) | 6 (21.4%) | 20 (36.4%) | 13 (29.9%) |
| *iceA* | Positive | 6 (20.4%) | 6 (22.4%) | 16 (29.1%) | 13 (28.6%) |
| **Virulence expression** |  |  |  |  |  |
| *cagA* expression | High | 0 (0.0%) | 3 (10.6%) | 31 (56.4%) | 15 (33.3%) |
| *vacA* expression | High | 2 (6.1%) | 6 (22.7%) | 23 (41.8%) | 14 (30.3%) |
| *babA* expression | High | 5 (15.0%) | 8 (30.0%) | 17 (30.9%) | 11 (25.0%) |
| *iceA* expression | High | 11 (33.9%) | 2 (6.5%) | 18 (32.7%) | 12 (27.4%) |
| **Clarithromycin resistance markers** |  |  |  |  |  |
| CLAR_*gyrA* | Positive | 8 (27.1%) | 2 (7.1%) | 1 (1.8%) | 29 (64.3%) |
| A2142G | Positive | 8 (24.4%) | 1 (4.4%) | 0 (0.0%) | 32 (71.1%) |
| A2143G | Positive | 6 (19.3%) | 2 (8.8%) | 0 (0.0%) | 32 (71.9%) |
| **Metronidazole resistance markers** |  |  |  |  |  |
| MET_*frxA* + *rdxA* | Positive | 5 (17.5%) | 2 (8.8%) | 23 (41.8%) | 15 (32.8%) |
| *frxA* | Positive | 4 (11.4%) | 1 (4.3%) | 31 (56.4%) | 13 (28.6%) |
| *rdxA* | Positive | 2 (6.0%) | 0 (0.0%) | 27 (49.1%) | 20 (45.0%) |
| **Levofloxacin resistance markers** |  |  |  |  |  |
| LEVO_*gyrA* | Positive | 5 (15.4%) | 6 (20.0%) | 22 (40.0%) | 12 (26.2%) |
| *gyrA*_AS59G | Positive | 2 (5.8%) | 0 (0.0%) | 24 (43.6%) | 23 (52.2%) |
| *gyrA*_AS131G | Positive | 3 (8.0%) | 0 (0.0%) | 26 (47.3%) | 20 (45.3%) |
| *gyrA*_261A | Positive | 9 (30.0%) | 0 (0.0%) | 17 (30.9%) | 18 (40.0%) |
| *gyrA*_272T | Positive | 5 (14.8%) | 9 (33.3%) | 19 (34.5%) | 8 (18.5%) |
| *gyrA*_272G | Positive | 3 (9.7%) | 2 (6.5%) | 25 (45.5%) | 17 (38.7% |

**Supplementary Table S11. Association between** *H. pylori* **molecular profiles and clinicopathological features in gastric cancer patients (n = 158)**

| **Feature** | **Category** | **Cluster 1: Low-Virulence Inflammatory (n = 31)** | **Cluster 2: Treatment-Sensitive Inflammatory (n = 27)** | **Cluster 3: Competent Colonizer (n = 55)** | **Cluster 4: Multi-Resistant Oncogenic (n = 45)** | ***p*-value** |
| --- | --- | --- | --- | --- | --- | --- |
| **Demographics** |  |  |  |  |  |  |
| Age (years) | Median (IQR) | 52 (40–65) | 55 (45–66) | 57.5 (45–66) | 48 (40–65) | 0.465 |
| Sex | Male | 19 (61.3%) | 13 (48.1%) | 34 (61.8%) | 21 (46.7%) | 0.316 |
|  | Female | 12 (38.7%) | 14 (51.9%) | 21 (38.2%) | 24 (53.3%) |  |
| Smoking | No | 21 (67.7%) | 19 (70.4%) | 33 (60.0%) | 31 (68.9%) | 0.768 |
|  | Yes | 10 (32.3%) | 8 (29.6%) | 22 (40.0%) | 14 (31.1%) |  |
| **Tumor Characteristics** |  |  |  |  |  |  |
| Clinical stage | Early (I–II) | 23 (74.2%) | 22 (81.5%) | 34 (61.8%) | 24 (53.3%) | 0.064 |
|  | Late (III–IV) | 8 (25.8%) | 5 (18.5%) | 21 (38.2%) | 21 (46.7%) |  |
| Histological type | Adenocarcinoma | 23 (74.2%) | 16 (59.3%) | 35 (63.6%) | 31 (68.9%) | 0.637 |
|  | Other | 8 (25.8%) | 11 (40.7%) | 20 (36.4%) | 14 (31.1%) |  |
| Differentiation grade | Well differentiated | 9 (29.0%) | 8 (29.6%) | 21 (38.2%) | 5 (11.1%) | **0.031** |
|  | Moderately differentiated | 4 (12.9%) | 5 (18.5%) | 14 (25.5%) | 17 (37.8%) |  |
|  | Poorly differentiated | 6 (19.4%) | 5 (18.5%) | 11 (20.0%) | 14 (31.1%) |  |
|  | Non-malignant / atypical | 12 (38.7%) | 9 (33.3%) | 9 (16.4%) | 9 (20.0%) |  |
| Tumor location | Distal stomach | 7 (22.6%) | 10 (37.0%) | 14 (25.5%) | 13 (28.9%) | 0.096 |
|  | Proximal stomach | 7 (22.6%) | 6 (22.2%) | 16 (29.1%) | 7 (15.6%) |  |
|  | Whole stomach / non-specific | 14 (45.2%) | 6 (22.2%) | 9 (16.4%) | 11 (24.4%) |  |
|  | Multi-organ / upper GI | 3 (9.7%) | 5 (18.5%) | 16 (29.1%) | 14 (31.1%) |  |
| **Infection Status** |  |  |  |  |  |  |
| *H. pylori* histology | Positive | 14 (45.2%) | 24 (88.9%) | 50 (90.9%) | 41 (91.1%) | **< 0.005** |
|  | Negative | 17 (54.8%) | 3 (11.1%) | 5 (9.1%) | 4 (8.9%) |  |
| **Clinical Outcomes** |  |  |  |  |  |  |
| Survival status | Deceased | 7 (22.6%) | 9 (33.3%) | 20 (36.4%) | 17 (37.8%) | 0.369 |
|  | Under treatment | 14 (45.2%) | 8 (29.6%) | 24 (43.6%) | 20 (44.4%) |  |
|  | Cured | 10 (32.3%) | 10 (37.0%) | 11 (20.0%) | 8 (17.8%) |  |
